# Chinese Primary School Students’ Peer Relationship and Chinese Language Scores: The Chain Mediation Effect of Parental Involvement and Sense of Autonomy

**DOI:** 10.3389/fpsyg.2022.738982

**Published:** 2022-03-21

**Authors:** Huiyan Qiu, Jiang Chai

**Affiliations:** ^1^Faculty of Psychology, Tianjin Normal University, Tianjin, China; ^2^School of Educational Science, Yancheng Teachers University, Yancheng, China

**Keywords:** peer relationship, parental involvement, sense of autonomy, chain mediation, Chinese language scores

## Abstract

This study investigated the internal mechanism of the relationship between primary school students’ peer relationships and their performance in the Chinese language and literature. We constructed a chain mediation model, focused on the mediation effects of parental involvement and the sense of autonomy, on the correlation between peer relationships and performance in Chinese language scores. A questionnaire survey was conducted among 1,503 students in grades 4–6, and their parents, in three cities in Jiangsu Province. The result indicated the following: (1) there was a significant positive correlation between primary school students’ peer relationships, parental involvement, sense of autonomy, and the level of Chinese language scores; (2) parental involvement and the sense of autonomy, respectively, mediate the relationship between peer relationships and Chinese language scores; (3) primary school students’ parental involvement and sense of autonomy play a chain-like mediating role in the relationship between their peer relationships and Chinese language scores. The research results provide a reference for exploring the educational strategies of primary school students’ Chinese literacy.

## Introduction

Academic achievement is a direct response to the students’ academic mastery level (Gijselaers et al., 2017; Stoeger et al., 2017; Madigan and Curran, 2021). Therefore, it is an important indicator for measuring and reflecting the status of learning, which has been the focus of schools and families. The subject of Chinese has an important place at the primary level. The increasing difficulty of the Chinese language curriculum, especially after the third grade in primary school, places higher demands on students’ reading, comprehension, and writing abilities. Many students lag in reading comprehension and analysis skills and are, therefore, academically disadvantaged. According to a survey, the detection rate of academically underachieving students in elementary school was 5.41% (Liu and Min, 2001).

Studies have found that the factors affecting academic performance consist of the individual and social environment (Lee and Shute, 2010). Peer relationships are an important influence on the academic performance of elementary school students (Chen et al., 2003; Gallardo et al., 2016). While many studies have shown that peer relationships positively predict students’ English achievement test scores (Phillips Galloway and Uccelli, 2019), little attention has been paid to the relationship between peer relationships and Chinese language scores. Although researchers have examined factors that may contribute to improved Chinese language scores, existing findings have focused on elementary school students’ memory (Peng et al., 2018), and reasoning (Sternberg et al., 2008). In contrast, previous studies paid less attention to the influence of autonomy on Chinese language scores. Instilling a sense of autonomy is a major goal of language education (Benson and Lor, 1998). In language acquisition, a sense of autonomy is the best predictor of language ability (Liu, 2015), as it enables students to acquire self-determination experience and control their learning ability. The social environment created by schools and families is an additional factor that cannot be ignored when considering Chinese language scores (Vasquez et al., 2016). Previous studies mainly focused on paternal parenting styles (Milevsky et al., 2007), family economic status (Sirin, 2005), and parental expectations (Rutchick et al., 2009). With the in-depth development of home–school cooperation, the influence of family education on academic performance has received increasing attention (Bæck, 2010). Current research on parental influence on language achievement is beginning to focus on the role of parental involvement (Niehaus and Adelson, 2014).

The above findings support the unilateral effects of peer relationships, parental involvement, and sense of autonomy on students’ academic performance, but fail to provide a systematic and comprehensive description of the underlying mechanisms that influence Chinese language scores. Concurrently, the existing literature provides no clear evidence of studies that have examined peer relationships and Chinese language scores’ chain mediation in the relationship between parental involvement, and a sense of autonomy. To bridge this gap, this study focused on the effect mechanism of peer communication, parental involvement, and sense of autonomy, on Chinese language scores, to provide strategies for improvement of such scores of primary school students.

## Literature Review

### Peer Relationships and Chinese Language Scores

As pupils’ independence and autonomy gradually grow, they become less dependent on their parents and begin to hold peer communication in the school environment (McDonald et al., 2010). Peer communication refers to a type of interpersonal relationship that is formed and developed through communication between individuals of the same age, or at the same level of psychological development (Zhang and Lin, 1999). Studies have shown that peer interaction is significantly related to language scores (Wentzel et al., 2010; Bellmore, 2011). Peer relationships have a greater impact on academic performance (Yang, 2008). Students with good peer relationships achieve more in their studies, whereas children who have suffered peer abuse in the third grade of elementary school, have poor academic performance after the fifth grade (Liu et al., 2014). Students’ acquisition of knowledge, methods, and the formation of emotional attitudes and values, are often accompanied by peer effects. Research has found that student performance is positively affected by peers. For every unit increase in peer reading scores, personal scores increase from 0.15 to 0.40 units (Hoxby, 2000). Based on previous studies, peer communication plays a crucial and decisive role in improving Chinese language scores. Therefore, we put forward the following hypothesis:

H1: The peer communication of primary school students is positively correlated with their Chinese language scores.

### Peer Relationships, Parental Involvement, and Chinese Language Scores

The family, as the first major environment for the development of primary school students, plays an extremely important role in their development (Brooks-Gunn et al., 2000). Parents have a subtle and long-term impact on their children. According to the theory of family capital, parents are involved in their children’s learning and can improve their academic performance by actively guiding and regulating their learning habits (Wei et al., 2016). Studies have found that parental involvement is a critical factor affecting students’ academic performance (Coleman, 1988; Lee and Bowen, 2006; Hill and Tyson, 2009). Parental involvement has a significant positive impact on children’s academic performance. More precisely, parent–child reading and parent–child communication can significantly improve children’s academic performance, with the latter having the greatest impact (Paquette et al., 2003; Wilson and Prior, 2011; Liu et al., 2020). Parental involvement has a significant impact on the Chinese language scores of students, especially disadvantaged and lower grade students (Li B., 2018).

In peer relationships, primary school students imitate and learn from the learning behaviors of their peers (Zhang et al., 2019). Peer learning behavior is divided into positive learning behavior and negative learning behavior. Studies have found that the more exposed the students are to negative learning behaviors, the more likely they are to exhibit negative learning behaviors. Such students may attract the attention of parents and teachers, thus increasing parental supervision, parent–child communication, and other parental involvement behaviors (Gao and Xue, 2020). In contrast, when students have more peers who engage in active learning behaviors around them, especially when their parents engage in more parent–child activities and communication, these students will ask their parents for more companionship and academic guidance, which can increase their parents’ involvement (Stewart, 2008).

To summarize, peer relationships are beneficial in promoting parents’ involvement. The higher the level of parents’ involvement, the higher the level of Chinese language scores, implying that parental involvement may serve as an intermediary between peer relationships and Chinese language scores. To study this prediction, we propose the following assumption:

H2: The relationship between peer relationships and academic performance is mediated by parental involvement.

### Peer Relationships, Sense of Autonomy, and Chinese Language Scores

A study by Lin (2017), about Chinese students’ core literacy revealed that the development of core literacy, “the all-round development of the people,” requires independent development, social participation, and cultural basis in three fields. Autonomy refers to a behavioral tendency of pursuing self-discipline of behavior; however, it is consistent with collective behavior goals. It is a sense of freedom that individuals experience in actively controlling the content and process of learning (Liu and Ling, 2009). Students’ sense of autonomy is an important intrinsic motivation that affects their academic performance and emotional adaptation (Grolnick and Slowiaczek, 1994; Levesque et al., 2004). Studies have suggested that unless students act autonomously in learning, it is difficult for them to have positive academic performance (Levesque et al., 2004). Only under the condition of independent support can students fully develop their abilities (Levesque et al., 2004; Skinner et al., 2009). The school motivation model found that students engage fully in learning activities only when the three basic psychological needs of children, namely, competency needs, autonomy needs, and belonging needs, are met (Ryan and Sapp, 2007). Students who achieve academic excellence have a higher sense of autonomy in their studies, whereas students with poor academic achievements have a lower sense of autonomy (Liu and Ling, 2009).

Learning in class is a type of interpersonal activity for students. Their participation in group communication, cooperative learning, and class discussion, can help them develop a sense of peer association, ability, and autonomy (Ryan and Deci, 2000). When students’ autonomous needs are supported by peers, they usually have a sense of choice and self-recognition of their behavior (Gagné and Deci, 2005; Deci and Ryan, 2008). Concurrently, students are more willing to participate and invest in learning activities. Studies have shown that promoting positive peer interaction is beneficial to the generation of primary school students’ learning enthusiasm and motivation. When students’ intrinsic motivation is improved, academic autonomy is easier to obtain (Reeve, 2012).

In summary, peer relationships are beneficial to students’ sense of autonomy, and students’ sense of academic autonomy is associated with the improvement of academic performance, indicating that autonomy may serve as an intermediary between peer interaction and Chinese language scores. We put forward the following assumption:

H3: The correlation between peer relationships and Chinese language scores is mediated by students’ sense of autonomy.

### Peer Relationship, Parental Involvement, Sense of Autonomy, and Chinese Language Scores

Birch and Ladd (1996) emphasized that both parental involvement and individual psychological characteristics (e.g., social cognition and sense of autonomy) have important effects on students’ learning, which indicated an important direction for studying the influencing mechanism of Chinese language scores. Parents’ involvement in learning can promote students’ positive self-representation and help them gradually realize that they are the leaders of learning. Accordingly, students can improve their academic performance under the condition of independent support (Skinner et al., 2009). Specifically, parental involvement in tutoring homework, has a positive predictive effect on students’ autonomous motivation of homework (Katz et al., 2011), and a positive predictive effect on students’ academic ability, learning motivation, and academic performance (Grolnick, 2015). In other words, parental involvement can positively predict students’ sense of autonomy (Deslandes et al., 1999). For primary school students’ sense of autonomy, parental involvement plays an important role in providing support for students’ psychological needs (Deci and Ryan, 2000). Therefore, parental involvement as an important aspect of the family environment can help stimulate students’ sense of autonomy and affect their academic performance.

A review of existing studies on the chain mediation model revealed that Mo et al. (2018) found the chain mediation of parental emotional warmth and psychological quality; Zhang et al. (2020) found the chain mediation of parental educational involvement and self-educational expectations, and Chen et al. (2021) found the chain mediation of parental involvement in students’ self-educational expectations. Although previous studies have also investigated the factors influencing academic achievement from the perspectives of parents and students, no studies have explicitly proposed a chain mediating role of parental involvement and sense of autonomy between parental relationships and Chinese language scores for the primary school student population.

H4: Parental involvement and a sense of autonomy are chain mediators in the relationship between peer leadership and Chinese language scores.

**FIGURE 1:**
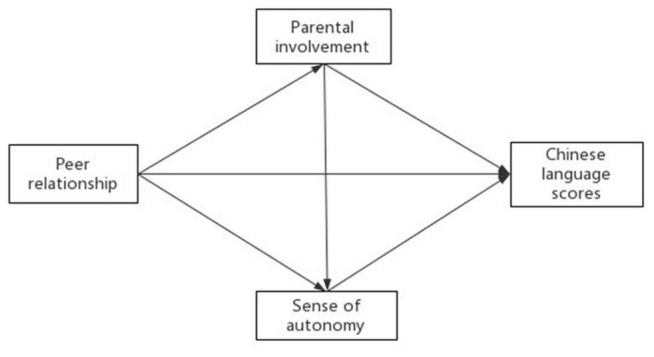
A hypothetical model of the relationship between variables. Peer relationship’s influence on Chinese language scores through Parental involvement and Sense of autonomy.

The specific path diagram is shown in Figure 1.

## Materials and Methods

### Sample and Data Collection

The stratified cluster sampling method was used to recruit students and their parents, from 12 primary schools, in three cities of Jiangsu Province. This is because the development scale, school-running level, and comprehensive strength of education in Jiangsu are among the best in China, and the 12 schools are evenly distributed in urban and rural structures. A total of 2,160 questionnaires were distributed to students; 1,800 were returned. Questionnaires with only certain parts completed were excluded; As a result, 1,503 questionnaires were finally valid, yielding an effective rate of 85.5% (age range = 9–13 years, Mage = 11.02 years, SD = 0.92; 50% female). In the valid questionnaires, there were 485 fourth graders (32.3% of the total), 490 fifth graders (32.6%), and 528 sixth graders (35.1%).

### Measures

#### Peer Interaction Questionnaire

The test was adapted from the Peer Interaction Questionnaire (Han, 2008), which measures the level of peer interaction. It consists of eight questions and is divided into two dimensions: cooperative learning and activity interaction. It uses a scale of 1–5 (1 = strongly disagree, 5 = strongly agree). After testing, the Cronbach α coefficient of the questionnaire was 0.923, that of the cooperative learning subscale was 0.829, and that of the activity interaction subscale was 0.783. The confirmatory factor analysis showed a good fit: χ^2^/df = 1.13, *P* > 0.05, RMSEA = 0.009, SRMR = 0.010, RFI = 0.994, GFI = 0.998, AGFI = 0.993, and NFI = 0.997. The questionnaire has good reliability and validity, is reliable, and the indicators of the questionnaire fit well.

#### Parent Involvement Questionnaire

The Parent Involvement Questionnaire was derived from literature (e.g., Epstein, 2001) and our knowledge of parental involvement in behavior, and it was used to assess the parent–child relationship. It consisted of 14 questions, divided into two dimensions of parent–child communication and parent–child activities, and scored from 1 to 5 (1 = strongly disagree, 5 = strongly agree). Parents were asked to explain their level of compliance with 14 participation behaviors. For example, items included, “I often discuss learning issues with my children,” and “I often take my children to participate in some social welfare activities.” Following testing, the Cronbach α coefficient of the questionnaire was 0.923, the coefficient of parent–child communication subscale was 0.902, and the coefficient of parent–child activity subscale was 0.908. The confirmatory factor analysis results showed that the fit was good: χ^2^/df = 1.23, *P* > 0.05, RMSEA = 0.012, SRMR = 0.014, RFI = 0.989, GFI = 0.999, AGFI = 0.988, and NFI = 0.994. The questionnaire has good reliability and validity, and the indicators of the questionnaire fit well.

#### The Sense of Autonomy Questionnaire

The Sense of Autonomy Questionnaire was adapted from the Barriers to Adolescents Seeking Help scale (Wilson and Deane, 2012). To assess the level of autonomy in primary school students, three dimensions of self-consciousness, self-management, and self-study were proposed, with 21 items and measured on a five-point Likert scale (1 = strongly disagree, 5 = strongly agree). Exemplary items are “I think I should work out my problems,” “I can make learning goals or plans that suit me,” and “I will take the initiative to preview new learning knowledge.” After testing, the Cronbach α coefficient of the total questionnaire was 0.93, and the coefficients of the three factors were between 0.78 and 0.86. Confirmatory factor analysis showed that the fit was good: χ^2^/df = 1.19, *P* > 0.05, RMSEA = 0.012, SRMR = 0.015, RFI = 0.980, GFI = 0.990, AGFI = 0.983, and NFI = 0.986.

#### Chinese Language Test Paper

According to the quality monitoring of primary school Chinese in Jiangsu Province, Li and Zhou (2012) proposed the main subjects of the Chinese subject, based on the results of the test and analysis (Wan, 2014), and as in the “Jiangsu Province Primary School Chinese Profession Quality Analysis Report.” The study serves as the theoretical basis. Based on the research results of text analysis, interviews, and expert evaluation, this research proposed the evaluation dimensions of primary school students’ language knowledge and skill literacy, which are divided into four parts: basic knowledge, understanding and application, reading, and writing. In a Chinese Language Test Paper, students are required to write Chinese characters according to Chinese pinyin, such as “duan lian.” With their consent, the students in grades 4–6 were tested collectively by the class as a unit, and the test was conducted by experienced graduate students, as the principal testers. During the test, the principal tester read the unified guidance and instructed the subjects to complete the questionnaire independently, which lasted for 80 min and was immediately returned after completion. The difficulty of the test papers for grades 4–6 is 0.653–0.724; the degree of discrimination is high, and additionally, the reliability of the expert consistency of the test paper is high.

### Data Analysis

SPSS 24.0 and AMOS 24.0 were used for the statistical processing of the data. SPSS 24.0 was used to test the common method deviation and examine the correlation between variables. AMOS 24.0 was used to construct the structural mediation models.

#### The Chain Mediation Model

In the chain mediation model, multiple mediating variables exhibit sequential characteristics and form a chain of mediators (Liu and Ling, 2009). First, AMOS 24.0 was used to construct the structural equation model. Chinese Primary School Students’ peer relationships were the independent variable (X), Chinese Language Scores were the dependent variable (Y), parental involvement was the first order mediator (M1), and sense of autonomy was the second-order mediator (M2). Second, the factor method was used to package the variables in this study (Wu and Wen, 2011). Finally, the Bootstrap program was used to test the effects of the chain mediation model. This model was implemented with 2,000 bootstrap samples and 95% corrected confidence intervals (CIs). If the 95% corrected confidence intervals do not include 0, the indirect effect is significant.

#### Common Method Bias Test and Correlation Analysis

We used Harman’s single factor test to check the common method bias. The results showed that a total of 11 common factors, with eigenvalues higher than 1 were proposed, and the first common factor explained 15.1% of the variances, which was less than 40% of the criterion proposed by Podsakoff (Zhou and Long, 2004), indicating that there was no serious common method bias in our study.

## Results

### Correlation Analysis

We conducted a correlation analysis to examine the association among all the variables, and the correlation matrix is illustrated in Table 1. Table 1 shows a significant positive correlation between the four variables of peer relationships, parental involvement, sense of autonomy, and Chinese language scores.

**TABLE 1:** Descriptive statistics results and correlation matrix.

Variable	1	2	3	4	5	6	7	8	9
1. Peer cooperation	1								
2. Interpersonal communication	0.703**	1							
3. Peer relationship	0.928**	0.918**	1						
4. Parental involvement	0.281**	0.311**	0.320**	1					
5. Sense of autonomy	0.638**	0.700**	0.724**	0.427**	1				
6. Self-consciousness	0.479**	0.501**	0.531**	0.395**	0.814**	1			
7. Self-management	0.585**	0.633**	0.659**	0.311**	0.851**	0.467**	1		
8. Self-study	0.568**	0.661**	0.664**	0.358**	0.868**	0.483**	0.778**	1	
9. Chinese language scores	0.170**	0.194**	0.197**	0.064*	0.209**	0.147**	0.210**	0.182**	1
*M*	4.14	4.06	4.10	3.87	4.18	4.40	4.12	3.89	64.10
SD	0.88	0.83	0.79	0.75	0.60	0.65	0.69	0.78	14.52

n = 1503; *p < 0.05; **p < 0.01.

### Mediation Analysis

According to the mediating effect test process proposed by Wen and Ye (2014), this study used a structural equation model to test the chain mediating effect of parental involvement and sense of autonomy, on peer interaction and Chinese language scores.

#### Mediation Analysis With Parental Involvement and Sense of Autonomy as Mediators

**FIGURE 2:**
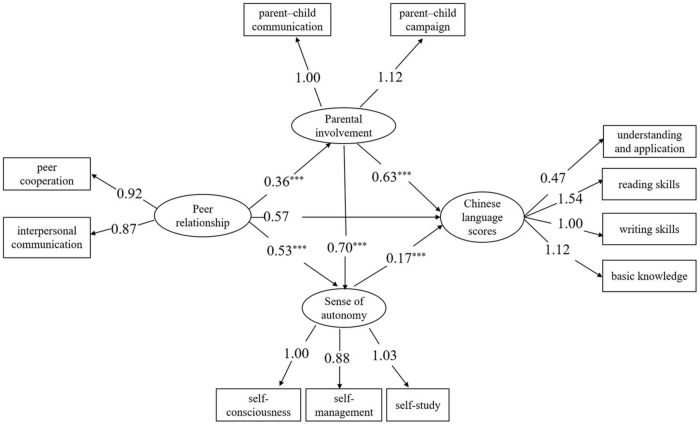
Model result. Chinese primary school students’ peer relationship and Chinese language scores: the chain mediation effect of parental involvement and sense of autonomy. ^***^p < 0.001; *n* = 1503.

Figure 2 shows a chain mediation model mediated by parental involvement and autonomy. According to the model fit results, the model fit well, χ^2^/df = 1.48, *p* > 0.05, RMSEA = 0.018, GFI = 0.998, NFI = 0.997, CFI = 0.999, IFI = 0.999. The results showed that the measurement model has reached the ideal standard, and the observed variables can better reflect the corresponding latent variables, which can further test the structural model.

For H1, we posited that peer relationships have a significant positive impact on Chinese language scores. However, the results showed that peer relationship has no significant direct predictive power on Chinese language scores (β = 0.57, SE = 0.33, *P* = 0.08). Therefore, H1 was not supported.

For H2, we proposed that parental involvement plays a mediating role in peer relationships and Chinese language scores of primary school students. The results showed that the path coefficient of peer relationships → parental involvement was significant (β = 0.36, SE = 0.03, *p* < 0.001), and parental involvement → Chinese language scores was also significant (β = 0.63, SE = 0.18, *p* < 0.001). The overall indirect effect of peer relationships → parental involvement, Chinese language scores was significant [β = 0.22, SE = 0.07, 95% CI (0.087, 0.381)]. Therefore, H2 was supported.

For H3, we proposed that a sense of autonomy plays a mediating role in peer relationships and Chinese language scores of primary school students. The results showed that the path coefficient of peer relationships → sense of autonomy was significant (β = 0.53, SE = 0.21, *p* < 0.001), and sense of autonomy → Chinese language scores was also significant (β = 0.17, SE = 0.05, *p* < 0.001). The overall indirect effect of peer relationships → a sense of autonomy → Chinese language scores was significant [β = 0.10, SE = 0.30, 95% CI (0.240, 1.419)]. Therefore, H3 was supported.

For H4, we assumed that parental involvement predicts the sense of autonomy, and they both play a chain mediating role in peer interaction and academic performance. The results show that the path coefficient of parental involvement → a sense of autonomy was significant (β = 0.70, SE = 1.56, *p* < 0.001), and the overall indirect effect of peer relationships → parental involvement → sense of autonomy → Chinese language scores was significant [β = 0.04, SE = 0.02, 95% CI (0.012, 0.100)]. Therefore, H4 was supported.

The indirect effect in the correlation between the peer relationships and Chinese language scores via parental involvement and sense of autonomy was significant, and the mediating variables explained 38.75% of the total variance (see Table 2). The results indicated that parental involvement and sense of autonomy played a mediating role in the relationship between the peer relationships and Chinese language scores.

**TABLE 2:** Effect sizes with parental involvement and sense of autonomy as mediators.

Effect	Indirect effect value	Point estimate (95% confidence interval)	Proportion
Total	0.36	0.32 (0.024, 1.266)	38.75%
PR→PI→SA→Chinese	0.04	0.02 (0.012, 0.100)	4.30%
PR→PI→Chinese	0.22	0.07 (0.087, 0.381)	23.70%
PR→SA→Chinese	0.10	0.30 (0.240, 1.419)	10.75%

*PR, Peer relationship; PI, Parental involvement; SA, Sense of autonomy; Chinese, Chinese language scores.*

## Discussion

By analyzing the mediation effect, this study found that peer relationships have no direct effect on students’ Chinese language scores, partly rejecting the first hypothesis (H1). This is in contrast to previous findings reporting that peer relationships positively predict students’ English achievement test scores (Wentzel, 1999). However, this result supports the findings concluded by Li D. (2018), who reported that there is no significant correlation between peer relationships and academic achievement.

Another result reveals that the relationship between peer relationships and Chinese language scores is mediated by parental involvement, which confirms the second hypothesis (H2). Through parental involvement, peer interaction influences academic achievement. The quality of peer relationships influences the level of parental involvement, and reciprocal peer quality does not only have a positive effect on individual academic performance, but also leads to the exchange and sharing of educational experiences among reciprocal peer parents (Lavy and Sand, 2011). Academic achievement is an extraordinarily valued factor in traditional Chinese culture (Liu et al., 2014). Children with higher academic performance are often not only admired by their peers (Zhou and Liu, 2014), but also by their peers’ parents (Phillipson and Phillipson, 2007). Parents whose children are academically underdeveloped will try to emulate the parenting styles of high–achievers’ parents, improve poor parenting practices, and learn to help their children pursue positive academic outcomes through encouragement, example, and emotional support (Patrick et al., 2004; Wentzel, 2005). Parents who are frequently involved in their children’s academic activities can encourage them to value their learning and hold high expectations through encouragement and praise. Thus, leading to positive and sustained attitudes toward language learning and contributing to children’s academic achievement.

Moreover, the mediation role of a sense of autonomy on the association between peer relationships and Chinese language scores is supported, thereby supporting the third hypothesis (H3). This result provides evidence for the previous theoretical arguments (Deci and Ryan, 2000). According to the “self-determination theory,” individuals tend to have active self-integration, self-improvement, and continuous learning (Liu et al., 2013). However, this tendency can only be realized through the support of external factors; peer communication represents critical social support for students’ academic adaptation at school. Good peer communication allows students to enhance their sense of autonomy (Ruzek et al., 2016). When students are more easily accepted by their peers and they cooperate more closely, they have a stronger desire to learn and self-manage. Furthermore, this result is consistent with previous empirical findings (Ryan, 2001; Choukas-Bradley et al., 2015; Sprenger and Guerra, 2018). Positive peer relationships can enhance students’ sense of autonomy, help learners to fully express themselves, and continuously adapt their actions in the process of individual–individual interaction (Zuo and Huang, 2008), and ultimately achieve common development in the learning community. The forms of peer discussion, mutual questioning, and division of labor and cooperation that arise under peer learning (Li et al., 2021), provide students with opportunities, to engage in role-play, select social perspectives, fully engage in accomplishing inter-individual peer cooperation and intra-individual cognitive coordination, and have autonomous control over classroom space and resources. Chinese is a subject with high requirements for “listening, speaking, reading, and writing,” which requires greater autonomy from students. When students are ready to communicate and cooperate, a virtuous cycle of “willing to learn, good at learning, and more willing to learn” develops (Ge, 2019).

Finally, the last hypothesis received support (H4). This study found that peer relationships have a chain-mediated effect on students’ Chinese language scores, through parental involvement and students’ sense of autonomy. This suggests that in addition to parental involvement and a sense of autonomy, which independently mediate the relationship between peer relationships and primary school students’ Chinese language scores, parental involvement can also indirectly affect the students’ language scores by influencing their sense of autonomy. This chain mediating effect study is an integration and extension of studies on parental involvement and sense of autonomy (Hong and Ho, 2005; Wang et al., 2018), and studies on the sense of autonomy and academic achievement (Covington, 1986; Schunk and Pajares, 2005). By linking parental involvement and sense of autonomy to peer relationships, the internal mechanisms at work in primary school students’ Chinese language scores were revealed more comprehensively. Parents’ involvement in their children’s learning, such as keeping track of their students’ learning dynamics, paying attention to confusion in learning, and helping their children to solve their learning problems, will influence children’s positive representation of the self (Xu et al., 2008), and awareness of their abilities (Zhang and Su, 2015). It would also help the children realize that to become active learners, they should be more actively involved in learning (Uji et al., 2014).

## Conclusion

This research constructed a chain mediation model, which can be understood as the influence mechanism of the peer relationships on primary school students’ performance in the Chinese language. The results of the study show that peer relationships play an intermediary role in the performance of Chinese language scores through parental involvement and students’ sense of autonomy. Consequently, this study extends the literature on Chinese learning in an important novel direction.

The results have educational implications. Firstly, children have more peers with active learning behaviors, which can provide them with more Chinese language learning support through parental involvement and sense of autonomy. Secondly, the results indicate the importance of family in primary school students’ Chinese language learning. Families can fully integrate their resources, provide emotional and material support for children’s learning, engage in active and effective learning supervision, take the initiative to increase time and quality with their children, and integrate cultural, activity, and practical education into family life. This will ensure the family becomes the second classroom for these students to learn the Chinese language. Finally, the results of the study suggest that educators should concentrate on strengthening students’ sense of autonomy, which is currently unstable among primary school students who have a strong passion for learning, but cannot proficiently plan and act on their learning. Educators can enhance pupils’ Chinese language scores through the independent development of their learning process, and teachers can provide students with emotional support through effective instruction or positive environments (Fast et al., 2010), thereby promoting student autonomy (Greene et al., 2004). The above findings provide guidelines for basic educators and parents on how peer relationships can effectively enhance Chinese language scores.

## Data Availability Statement

The raw data supporting the conclusions of this article will be made available by the authors, without undue reservation.

## Author Contributions

HQ and JC developed the study concept, designed the research, and edited the manuscript. HQ performed the statistical analyses and drafted the manuscript. Both authors read and approved the final manuscript.

## Conflict of Interest

The authors declare that the research was conducted in the absence of any commercial or financial relationships that could be construed as a potential conflict of interest.

## Publisher’s Note

All claims expressed in this article are solely those of the authors and do not necessarily represent those of their affiliated organizations, or those of the publisher, the editors and the reviewers. Any product that may be evaluated in this article, or claim that may be made by its manufacturer, is not guaranteed or endorsed by the publisher.
